# A Biopsychosocial Approach to Grief, Depression, and the Role of Emotional Regulation

**DOI:** 10.3390/bs11080110

**Published:** 2021-08-04

**Authors:** Cristina Peña-Vargas, Guillermo Armaiz-Peña, Eida Castro-Figueroa

**Affiliations:** 1 Ponce’s Research Institute, Ponce Health Sciences University, Ponce, PR 00716, USA; 2 School of Medicine, Ponce Health Sciences University, Ponce, PR 00716, USA; garmaiz@psm.edu; 3 School of behavioral and Brain Sciences, Ponce Health Sciences University, Ponce, PR 00716, USA; ecastro@psm.edu

**Keywords:** grief, depression, psychopathophysiology, psychoneuroimmunology, affective neuroscience

## Abstract

According to the field of affective neuroscience, grief has been identified as one of the seven primary emotions necessary for human survival. However, maladaptive grief could cause significant impairment in an individual’s life, leading to psychopathologies such as major depressive disorder. Research on grief has shifted to a biopsychosocial approach, leaving behind outdated models—such as the Kübler-Ross theory—that have shown poor consistency. The field of psychoneuroimmunology has identified adverse life events such as social loss as being associated with major depressive disorder, and inflammatory processes in chronic health conditions. Likewise, scientists in the field of affective neuroscience have theorized that prolonged and sustained activation of the grief neurological pathway can cause a cascade of neurotransmitters that inhibits the reward-seeking system, causing symptoms of depression. The objective of this review is to highlight findings on the grief process using a biopsychosocial approach to explore grief’s impact on psychopathophysiology.

## 1. Introduction

Grief is a phenomenon that carries an unpleasant feeling, which human beings inevitably experience. Essentially, grief is the feeling or reaction manifested due to bereavement and mourning after a significant loss [1]. Grief can be stratified into several types, such as acute grief, adaptation to loss, and complicated grief [2]. However, in all of its gradients, grief seems to be an unwanted and unpleasant feeling that cannot be escaped. This is not to say that all individuals struggle while coping with grief, or that they do not reap virtues out of this process [3]. Bagbey Darian proposes a model of adaptive grieving dynamics that underlines how individuals can cope in healthy ways, and how some render personal growth out of the process [4]. Likewise, the field of affective neuroscience has stated that grief is essential for human survival (as it is for other mammals) [5]. According to a growing body of literature in the field of affective neuroscience, grief has been identified as a primary emotion necessary for maintaining social bonds [5]. However, in some instances, the grieving process can cause significant impairment and anguish in an individual’s life, leading to psychopathologies such as prolonged grief disorder or major depressive disorder [6], and could also lead to increased risk of suicidality [7]. Kübler-Ross model [8] described grief as a process with a set of stages (denial, anger, bargaining, depression, and acceptance), and has been widely used in the treatment and research of bereavement around the world. Nevertheless, research has shown poor consistency and validity concerning the linearity of these stages [9], considering this model to be outdated. In recent years, the Kübler-Ross model has continued to receive growing criticism [10,11,12]. 

Grief has been studied in the field of psychology due to its impact on mental health, causing distress and impairment of functionality for some individuals. As such, the ICD-11 and the DSM-5 include the diagnosis of prolonged grief disorder (ICD-11) or persistent complex bereavement-related disorder (DSM-5), respectively [13,14]. Both of these clinical manuals illustrate the distinctive clinical features of impairment caused by prolonged grief in contrast to other mental health disorders, such as major depressive disorder (MDD) or post-traumatic stress disorder (PTSD). Although the literature shows that maladaptive grief is distinctive from other psychopathologies, such as major depressive disorder and post-traumatic stress disorder [15,16,17], studies show that grief and loss are associated with these disorders [18,19,20]. However, literature on grief and bereavement poses some incongruences regarding what types of loss could trigger a grieving process or psychopathologies. Some studies show that non-social loss (e.g., loss of house, job, or health) can trigger a maladaptive grieving process [21,22,23,24,25]. Nevertheless, other studies have failed to find a statistical significance linked to it [26]. This may be because the Kübler-Ross model has been extensively used to conceptualize grief in research and treatment, despite its lack of empirical validity. 

Research and clinical practice have been changing directions in the conceptualization of grief, so as to accommodate research-informed models [27]. Likewise, some researchers have shifted their focus to a biopsychosocial approach for conceptualizing grief, in order to underline the importance of holistic approaches to studying grief, and its impact on psychopathologies [28,29,30]. This review will address two models with biopsychosocial frameworks for which Table 1 contains a summary. Slavich and Irwin developed a social signal transduction theory of depression, in which social loss and social rejection are linked to predictors of major depressive disorder [31]. They also linked social loss and social rejection with the onset or exacerbation of other major inflammatory health conditions that involve inflammatory processes (e.g., rheumatoid arthritis, chronic pain, obesity, diabetes, and cardiovascular disease) [31]. On the other hand, the field of affective neuroscience has explored social loss by conducting studies in animal models, which have helped advance understanding of the role of grief as one of the primary emotions [32]. Studies in the field of affective neuroscience have shed light on the impact of grief on depression, explaining why depression hurts [33]. The objective of this review is to highlight findings on the grief process using a biopsychosocial approach to explore its implications on major depressive disorder and pathophysiology associated with inflammation processes.

## 2. Loss and Grief: How Are They Associated with Prolonged Grief and Depression? 

There is a particular nuance in how individuals perceive loss and death and how each person copes with grief. There are various risks and protective factors that influence grief management [34]. Likewise, culture impacts the manifestation of the grief process, as collective beliefs about grief management, beliefs surrounding death, and psychological healing processes vary between cultures [35,36,37]. Similarly, the concepts of grief and loss can also be impacted by family values, dynamics, and systemic processes [38,39,40,41]. As grief has a phenomenological component to it, grief could be understood and manifested differently throughout the stages of life [42]. The meaning and concept of death is something that changes through different life processes [43,44]. Clinicians in a study were asked to state the difference between complicated grief and normal grief in children, and complicated grief seemed to be highlighted more by traumatic thoughts, self-blame, anger, and lack of safety in comparison to adults [45]. As manifestations of grief could vary, it is important to study predictors of grief identifying different types of loss and initial reactions to loss.

Although there are clinical differences between depression and prolonged grief as disorders, the death of a loved one could trigger not only grief, but also symptoms of depression. A study on perinatal loss showed that symptoms of both grief and depression were present in mothers during the first month after a loss [46]. Another study showed that 35.5% of the participants showed prolonged grief disorder (PGD) and MDD symptoms after an unnatural or violent death [47]. On the other hand, loss could also be studied through the loss of social support. Lower levels of social support have been correlated with poor grief outcomes and complicated grief in bereaved cancer patient caregivers [48]. Congruently to these findings, perceived lack of social support has been shown to be associated with MDD after a social loss [49]. As an example, a study conducted with university students who had encountered a significant loss showed that participants with lower perceived social support had higher prolonged grief disorder and depression symptoms [50]. These studies highlight the importance of social bonds. Social support has certainly been identified as a protective factor against depression’s symptomatology [51,52,53,54]. Although the source of social support could vary throughout a lifetime [55], as individuals give different meanings to social bonds, one example of an important bond is friendship. The loss of a friendship could be another loss to mourn [56]; however, there is scarce research on this area. Social bonds could be the cause of grief or a protective factor during the grieving process to prevent further complications in psychopathology, such as MDD, which is why it is of utmost importance to continue research in this area.

Existing literature has delineated that death is not the only type of loss that could trigger a grief process or psychopathology. Terminations of romantic relationships, such as a break-up or a divorce, are also forms of social loss from which individuals could experience grief [57,58,59]. Inclusively, in the context of romantic break-ups, a study showed how grief manifested differently between sexes—while women experienced more emotional responses, men exhibited more somatic responses [60]. Moreover, a study showed that the end of a romantic relationship was associated with both complicated grief and depression [61]. This investigation shed light on how the loss of a relationship could trigger both a prolonged grieving process and depression. Likewise, a study that developed an instrument for measuring yearning found that it was a significant factor during the grieving process after the death of a loved one, a romantic breakup, or homesickness [62]. Such findings could imply that a grieving process where yearning is present could be experienced after a social loss related not to death, but to a change in a relationship dynamic.

Nevertheless, social loss is not the only type of loss. Individuals can experience loss of conditions that could symbolize or be tied directly to a sense of security. An example of this could be job loss. Literature concerning grieving processes and job loss has been divergent; while some studies have shown a correlation with grief [63], others have not [64]. However, studies show a strong association between job loss and depression [65]. An investigation among older workers (55–65 years old) showed that job loss was a significant predictor of depression [66]. Another study showed that job loss could be associated with both depression and prolonged grief [67]. Although grieving the loss of a job could be part of the experience for some individuals, a relationship with psychopathology seems to be more consistent with job loss. This could be due to the vulnerabilities a job loss can pose for an individual, due to the numerous security and life factors that come into play. For example, financial strain is a mediating factor for depression after a job loss [68].

Another example of a type of loss is health loss. The diagnosis and adjustment to an illness convey multiple changes in an individual’s life, placing a person in a vulnerable position. Health loss can translate to the loss of a person’s usual functionality, and poses a lifestyle change that could be harrowing. A symbolic loss, such as hair loss for women with cancer or sexual dysfunction for men with prostate cancer, has been shown to have mental health repercussions [69,70]. Psychological factors associated with the grieving process have been identified in individuals who suffer from chronic conditions, in a study that incorporated the Kübler-Ross grief stages to the adaptation process [71]. This study on type 2 diabetes patients concluded that participants who accepted their condition had better management of their glycemic levels than participants the in denial or depression stages [71]. Similarly, a study using the Kübler-Ross model on patients with visual impairment showed that acceptance was associated with the patient’s wellbeing, while denial was associated with depression [72]. As stated before, these studies employing the Kübler-Ross model could be questioned on how accurately they may be exploring the experience of grief, due to the lack of empirical consistency of the stages model. Nevertheless, literature has shown a more tangible link between depression and health loss, suggesting that the probability of depression increases with the severity or number of chronic physical conditions that a person may have [73]. Additionally, depression could be associated with other biological factors, such as systemic inflammation, which could have a negative impact on chronic diseases [73].

**Figure 1:**
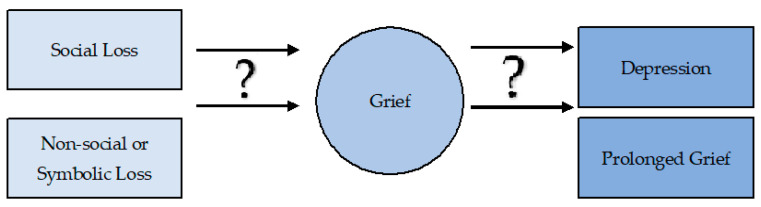
This model underlines the mediating factor that should be in question to determine associations between types of loss, grief, and psychopathologies.

Moreover, a study on transgender men and women examined the relationship between transition status, perception of loss, social support, and coping (facilitative and avoidant coping styles) with mental health outcomes such as depression and anxiety [74]. This study explored multiple types of losses (employment, housing, finances, healthcare, parental, sibling, romantic partner, children, and friends). Results showed that lack of social support and an avoidant coping style was directly and indirectly associated with depression. Nevertheless, loss was neither directly nor indirectly associated with depression. In summary, existing literature fluctuates between findings of non-social or symbolic loss and its relationship with prolonged grief and depression, making it of utmost importance to conduct further research using validated frameworks, methods, and instruments to ensure reliable results. There is a gap in knowledge yet to be address about what are the underlining factors or mechanism that could potentially influence in a transition between type of losses and grief. In addition, as grief and depression are so closely related, it would be ideal to define the role of grief as a mediating factor between loss and MDD. Figure 1 illustrates a model presenting the possible knowledge gaps. 

## 3. Neuro-Affective Pathways and Animal Models of Depression Linked to Grief

Phenotypic expression of loss can manifest in different manners. However, which physiological changes occur when a person experiences a loss? According to the field of affective neuroscience, seven neurological pathways have been identified across all mammals, which mediate basic emotions [5]. Primary emotions are the first reaction to any situation, and are located in primitive parts of the brain [75]. Emotions activate critical neurological pathways that are necessary for survival. In this next section, we will summarize important neuro-affective pathways that have been linked to grief and depression [32,76,77].

The PANIC/GRIEF system is one of the seven neuro-affective systems that mediate pathways needed for survival. Studies have shown that the PANIC/GRIEF system is responsible for feelings of sadness, despair, and panic when humans experience social loss [78]. Studies in rats have shown that the neurological grief pathway activates after mother–offspring separation [79]. In a study, rats were observed to make a call after maternal separation to seek help and prevent danger [79]. Grief is one of the first emotional reactions after a social loss, and the feeling of despair that grief causes has been associated with why depression “feels so bad” [80]. To study social bonds, one of the earliest studies in the field of affective neuroscience showed decreased mobility during the FST in young mice kept separated from their mothers [76]. In this study, immobility in the forced swimming test was conceptualized by the authors as despair and hopelessness.

Panksepp and Watt conceptualized grief as the feeling of despair caused after a social loss. On the other hand, the GRIEF system’s primary function is promoting social bonding [80]. This pathway is part of a group described as the negative effects that play a key role in supporting mammals’ survival instincts [5]. According to studies, electrical stimulus of the brain (ESB) mapping of the separation distress (GRIEF) system has highlighted a pathway from the dorsal periaqueductal gray matter (PAG) to the anterior cingulate cortex, which is aroused by glutamate and corticotropin-releasing factor (CRF) [81]. Two other neuropeptides involved in this circuit are oxytocin and prolactin, which inhibit the GRIEF system [80]. Oxytocin and prolactin are major social attachment and social bonding pathways in the mammalian brain [80]. Therefore, when these neuropeptides are present at high levels, they diminish the feeling of separation distress.

The other neurological system involved in Panskepp and Watt’s neuro-affective theory of depression is the SEEKING system. This neurological pathway is associated with reward-seeking, motivation, and anticipation in general [5]. This system is responsible for the impulse that drives mammals to look for basic and non-basic needs [5]; it is also of utmost importance because it guides the other primary emotions [5,32]. The SEEKING pathway system is dopamine-driven, and courses from the ventral midbrain to the nucleus accumbens and the medial frontal cortex [32]. Panksepp and Wright created a framework discussing the importance of the SEEKING system concerning its association with the conscience, and with different psychopathologies such as depression, addiction, and psychosis [82]. Congruently with these findings in the field of affective neuroscience, a study using the depression model of genetically modified rats called the Flinders sensitive line (FSL) found that isolation in these genetically modified rats provoked more anxiety than in a control group [83]. In this study, FSL rats showed decreased mobility in the forced swimming test compared to their control group, which is a measurement in animal models for anhedonia. However, the two groups displayed equal impairment of object recognition memory after isolation. The authors concluded that social isolation affected both groups of rats. However, these behavioral changes were strain-specific.

On the other hand, an animal study explored the impact of isolated rearing in weaning rats. This study highlighted the possible role of imbalances on dopaminergic pathways in psychological disorders, such as psychosis [84]. The authors found that isolation produced a range of persistent behavioral changes in young adults, including hyperactivity in response to novel stimuli [84]. Data from this study indicated an association with alterations to central aminergic neurotransmitter function in the mesolimbic areas. The investigators observed a series of neurochemical imbalances, which they hypothesized contributed to the exaggerated response of the isolated rats to novel stimuli, or to stimuli predictive of danger. The results of this study indicated that these isolation-reared rats presented enhanced presynaptic dopamine and 5-HT function in the nucleus accumbens, which was associated with decreased presynaptic 5-HT function in the frontal cortex and hippocampus.

However, 5-HT plays an important role in the expression of depression disorders [85,86]. Dopamine pathways could play a role in anhedonia due to dopamine’s principal role in the reward system [87]. When assessing anhedonia in a rat model, a study showed that tissue samples from the medial prefrontal cortex (mPFC), ventral tegmental area (VTA), and nucleus accumbens exhibited basal dynorphin levels that were similar to those seen in normal animals [88]. The study showed that orexin was reduced in the VTA and mPFC. The authors of this study also found that dynorphin and orexin were both diminished in the hypothalamus, which is noteworthy, since nearly all hypothalamic orexin cells co-express dynorphin. These findings suggest that orexin and dynorphin function may be imbalanced between the hypothalamus and the mesocortical/dopaminergic brain regions in depression.

Although social loss has been explored in rodent animal models using isolation paradigms, non-social loss (e.g., job, health, and house, among others) is a challenge to investigate in rodent animal studies and translate to human behavior. This is due to tertiary processes responsible for complex mind processes, which have a role in the integration of information in our neocortex—something explored so far only in humans [89]. Humans can assign meaning to phenomena, whereas there is no evidence of this so far regarding rodents. As scientists have yet to determine how non-social loss could be measured in rodent models, it is of key importance to conduct affective neuroscience research in human subjects, so as to understand the implications of loss in psychopathologies.

## 4. Loss, Grief, Depression, and Inflammatory Markers

Recent literature has explored different biomarkers that could be linked to inflammation, and their association with chronic psychological disorders [90]. This section will discuss recent literature that suggests that loss could lead to psychopathology, while at the same time having an impact at the physiological level. As grief could signify a stressful and even traumatic event, it has been studied to examine its physiological effect. A study that measured levels of inflammation markers and cortisol in relation to bereavement status and the number of recent bereavements showed that bereaved subjects had higher levels of inflammatory biomarkers [91]. This study assessed levels of interleukin 6 (IL-6), C-reactive protein (CRP), soluble intercellular adhesion molecule-1 (sICAM-I), soluble E-selectin (sE-selectin), and cortisol. Bereaved participants had higher levels of IL-6 and sE-selectin, but not CRP or sICAM-I, in comparison with the non-bereaved participants. Interestingly, the results from this study also showed that the number of recent bereavements was associated with higher levels of inflammation.

The type and closeness of the relationship should also be a factor taken into consideration in bereavement studies, as spousal bereavement is associated with enhanced pro-inflammatory cytokine production [92]. Moreover, increased levels of pro-inflammatory cytokines such as IL-6 and TNF-α have been shown to have a relationship with social disconnection and depressed mood [92], meaning that not only loss through the death of a loved one, but also loss of social connections, could have implications for inflammatory dysregulation. Congruent with these findings, one study showed that social support and social integration were related to lower levels of inflammatory factors such as IL-6 and CPR [93], which suggests a connection with social bonds and inflammatory reactions. On the other hand, research on cancer patients suggests that lack of social support could predict the development of pain, depressive symptoms, and inflammatory factors (e.g., IL-6 levels) [94]. This compilation of literature suggests that lack of social support could increase inflammatory factor levels, and a dysregulated grieving process could play a role in exacerbating inflammatory conditions.

Likewise, a study on bereaved individuals showed that participants with higher grief severity had greater levels of the inflammatory cytokines IFN-γ, IL-6, and TNF-α than those who showed less grief severity [95]. The results from this study also showed that patients with higher levels of depression presented elevated levels of inflammatory cytokines compared to participants who had lower levels of depression. This study suggests that patients with chronic inflammatory diseases could be at risk for exacerbated symptomatology. Furthermore, when exploring social loss from a change-in-dynamic point of view, a study on romantic breakups indicated that romantic ruptures might lead to symptoms of bereavement, including intrusive thoughts and attempts to suppress them, insomnia, and morbidity factors including broken heart syndrome and immune dysfunction [96]. Results from this study stated that compromised immune function could result from the reduced vagal activity and an increase in cortisol levels and catecholamines, leading to increased levels of inflammatory cytokines and decreased natural killer cell activity.

These studies shed light on the possible impacts that different types of social loss could have, leading to a dysregulation of inflammatory factors and potentially affecting individuals with chronic conditions. As the human body and mind are highly complex, there is also evidence that inflammation could cause psychosocial distress [97], suggesting a cycle of symptoms. For example, a study on patients with cardiovascular disease (CVD) showed that the patients with MDD and CVD had greater immune abnormalities, which could increase MDD symptoms, indicating a bidirectional relationship [98]. Nevertheless, there is a severe lack of studies regarding non-social loss, symbolic loss, or non-death-related grief. No studies were found during this review that assessed the potential association between non-death-related grief and inflammatory biomarkers. This lack of research underlines a knowledge gap that should be addressed in the efforts at improving the quality of life for patients with inflammatory conditions.

## 5. Grief, Emotional Regulation, and Inflammatory Biomarkers

A novel alternative to discover currently unknown pathways contributing to the SEEKING reward systems’ inhibition could be achieved by studying the phenotypic expressions (e.g., emotional regulation techniques) and, thus, determine the pathopsychophysiological mechanisms by explaining the potential transition from a maladaptive grieving process to clinical depression. Emotional regulation is a factor that merits investigation as a possible link between the two systems (PANIC/GRIEF and SEEKING), considering its role as a protective factor during stressful events [99]. According to Gross, emotional regulation is an individual’s ability to influence their emotions and the way in which they express their feelings [100]. That being said, the individual’s ability to regulate their grief could be a key factor in preventing depression. A study on a sample of relatives who had lost a loved one explored whether there was a relationship between self-compassion, prolonged grief, depression, and post-traumatic stress, and found that self-compassion was negatively associated with PGD, MDD, and PTSD levels [101]. This same study showed that grief rumination significantly mediated an association of higher self-compassion with lower depression and PTS. These findings suggest that people with higher self-compassion, which is a form of self-soothing, are less susceptible to experiencing psychopathology. Regarding the association of emotional regulation difficulties with neuroticism, avoidant attachment, and prolonged grief symptoms, a study showed that emotional regulation mediated the link between anxious attachment and prolonged grief symptoms [102]. The results of this study indicate that emotional regulation functions as a vehicle to manage attachment styles, which could serve as a protective factor against prolonged grief symptoms.

The findings from the studies above point to a very strong association of emotional regulation as a mediator of mental health outcomes during grief. Emotional regulation has been shown to have an impact on inflammatory reactivity [103]. A study aiming to investigate biomarkers related to emotional regulation showed that higher scores for the emotional regulation style called positive dyadic coping (couples working together to manage stress) were significantly associated with lower circulating levels of the inflammatory marker CRP [103] Additionally, this study showed that negative dyadic coping was unrelated to CRP. However, exploring emotional regulation’s role in inflammation reactivity during the grief process is important to identify potential protective factors against the exacerbation or progression of chronic conditions associated with inflammation processes.

Another example of a study linking emotional regulation’s role in mental health and physiology showed a potential impact on regulating inflammatory factors in bereaved spouses [104]. The results of this study indicate that spouses who reported suppressing their expression as a strategy for emotional regulation had a more pronounced inflammatory response. The authors measured inflammatory response using levels of a composite cytokine index consisting of interleukin (IL) 17A, IL-2, IL-6, tumor necrosis factor α, and interferon-γ. Likewise, a study aiming to examine a population of adults’ emotional regulation strategies and their relationship with inflammation showed that reappraisal was associated with significantly lower C-reactive protein (CRP) levels. In contrast, suppression was associated with significantly higher CRP [105]. This literature helps visualize emotional regulation as a protective factor against dysregulation of inflammatory factors in patients with inflammatory conditions or conditions that involve some inflammatory process as part of their treatment, such as cancer.

## 6. Conclusions

This review highlights the importance of social bonds. Social loss seems to be more consistently connected to grieving and depression than non-social losses or symbolic losses. However, there is a lack of research regarding the role of non-social or symbolic loss (non-death related), grief, and inflammatory biomarkers. Non-death-related loss could be a stressful situation potentially impacting patients with chronic inflammatory conditions. Patients with inflammatory conditions undergo a series of changes that could imply several types of losses, which should be assessed as risk factors. Discrepancies in the literature regarding the grieving process, its manifestation, and its relationship with different types of losses could be due to the use of unempirical models, which impact research outcomes. Therefore, it is of utmost importance to explore empirical models of grief and loss using validated measures, and research informed theories to understand this phenomenon in a more comprehensive manner.

According to a handful of the studies discussed below, loss could be a cause for major depressive symptomatology and dysregulation of immune processes. However, the underlying biopsychosocial factors that mediate these relationships need to be further explored in order to pinpoint the specific mechanisms that are taking place. The Kübler-Ross model, although widely used, poses inconsistencies, and as critics have exposed during the latest years, efforts to continue studying biopsychosocial bases for a grieving model should continue. However, as stated by Kübler-Ross, it might not be coincidental that depression is mentioned as one of the final stages of the grief process, as studies in affective neuroscience have delineated a relationship between grief and MDD. On the other hand, studies show that emotional regulation could play a part in coping with grief and loss, which may have an impact on the outcome of emotional distress and triggering psychopathologies.

During the process of this review, a variable not initially considered was attachment style. One of the studies regarding emotional regulation highlighted attachment styles. and showed a relationship with grief coping, consistent with Panksepp’s neuro-affective model. The GRIEF system’s role in social bonds can shed light on how attachment could be associated with coping styles and emotional regulation. It is crucial to develop empirical studies that can answer specific questions about the link between grief, depression, and physical health, with inflammatory conditions posing a more significant risk for symptom exacerbation. The field of affective neuroscience has identified key physiological pathways of primary emotions that can help us to understand how biology impacts emotions and the body. Using this information about the functions of primary emotions could guide researchers to better understand the endophenotypic expression of psychopathologies.

## Figures and Tables

**Table 1:** Biopsychosocial approaches to loss associated with depression.

Model	Authors	Theory
Affective neuroscience approach to depression	Jaak Panksepp and Douglas Watt	Sustained and prolonged activation of the GRIEF system leads (by a yet-unknown mechanism) to inhibition of the reward-seeking system. Inhibition of the reward-seeking system in rats manifests as surrender and defeat in different tasks (used to measured depression phenotype), which Panksepp and Watt conceptualized as anhedonia—one of the primary symptoms of major depressive disorder (MDD).
Social signal transduction theory	George Slavish and Michael Irwin	Prolonged early stress (such as loss) is a predictor of MDD and dysregulation of inflammatory processes in chronic health conditions.

## Data Availability

Not applicable.
